# Recurrent Skin and Lung Infections in Autosomal Dominant Hyper IgE Syndrome with Transactivation Domain STAT3 Mutation

**DOI:** 10.1155/2014/136752

**Published:** 2014-03-05

**Authors:** Chad J. Cooper, Sarmad Said, German T. Hernandez

**Affiliations:** Department of Internal Medicine, Texas Tech University Health Sciences Center, 4800 Alberta Avenue, El Paso, TX 79905, USA

## Abstract

*Background*. Hyper IgE is a rare systemic disease characterized by the clinical triad of high serum levels of IgE (>2000 IU/mL), eczema, and recurrent staphylococcal skin and lung infections. The presentation of hyper IgE syndrome is highly variable, which makes it easy to confuse the diagnosis with that of severe atopy or other rare immunodeficiency disorders. * Case Report*. A 23-year-old Hispanic presented with history of frequent respiratory and gastrointestinal infections as a child and multiple episodes of skin and lung infections (abscess) with *Staphylococcus aureus* throughout his adult life. He had multiple eczematous lesions and folliculitis over his entire body, oral/esophageal candidiasis, and retention of his primary teeth. The IgE was elevated (>5000 IU/mL). Genetic mutation analysis revealed a mutation affecting the transactivation domain of the STAT3 gene. * Conclusion*. The hallmark of hyper IgE syndrome is serum IgE of >2000 IU/mL. Hyper IgE syndrome is a genetic disorder that is either autosomal dominant or recessive. A definite diagnosis can be made with genetic mutation analysis, and in this case, it revealed a very rare finding of the transactivation domain STAT3 mutation. Hyper IgE syndrome is a challenge for clinicians in establishing a diagnosis in suspected cases.

## 1. Introduction

Hyper IgE is a rare systemic disease characterized by the clinical triad of high serum levels of IgE (>2000 IU/mL), eczema, and recurrent staphylococcal skin and lung infections [1]. It was first described as Job's syndrome in 1966, in patients suffering from recurrent sinopulmonary infections and cold skin abscesses due to *Staphylococcus aureus* [1]. The term was changed to hyper IgE syndrome, when an elevated level of IgE was discovered in these affected patients. There is no predilection for a certain gender or race. In recent years, the pathophysiology has been revealed through understanding the genetic components and consequences of the underlying condition. Hyper IgE syndrome is a complex immune deficiency with diverse clinical manifestations and heterogeneous genetic origins [2]. The presentation of hyper IgE syndrome is highly variability, which makes it easy to confuse the diagnosis with that of severe atopy or other rare immunodeficiency disorders.

The majority of patients with hyper IgE syndrome suffer from recurrent staphylococcal infections that predominantly involve the skin and lungs. Other features include abnormalities of the musculoskeletal system, hypermobility of the joints, prominent forehead, broad nasal bridge, macrocephaly, retention of primary teeth, and recurrent skin or respiratory infections [2]. Fungal infections, including mucocutaneous candidiasis and pulmonary aspergillosis, are also common. Patients with hyper IgE syndrome commonly have atopic dermatitis associated with very high levels of IgE and eosinophilia. But they do not have allergic manifestations, such as allergic rhinitis, asthma, urticaria, and anaphylaxis [3]. Hyper IgE syndrome has been associated with an increased risk of autoimmune diseases such as systemic lupus erythemathosus (SLE), dermatomyositis, and membranoproliferative glomerulonephritis [3]. These patients have an increased incidence of lymphoproliferative disorders such that non-Hodgkin and Hodgkin lymphomas have also been noted. Skeletal abnormalities include osteopenia, minimal trauma fractures, and scoliosis. Vascular abnormalities include middle-sized artery tortuosity and aneurysms, with infrequent clinical sequelae of myocardial infarction and subarachnoid hemorrhage.

The respiratory infections are most commonly caused by *Streptococcus pneumonia*, *Staphylococcus aureus,* or *Haemophilus influenzae* [4]. Pneumonias are usually complicated by lung abscesses, bronchopleural fistulas, bronchiectasis, and the formation of pneumatocele [4]. These bronchopulmonary lesions are predisposing factors for colonization by opportunistic microorganisms such as *Aspergillus fumigatus* and *Pseudomonas aeruginosa*. These pulmonary complications lead to the development of chronic respiratory insufficiency which is the main cause of mortality in hyper IgE syndrome [5]. Lung abscess with hemoptysis and cystic lung disease are other common causes of death in hyper IgE syndrome.

Characteristic oral and dental manifestations in hyper IgE syndrome include the delayed loss of primary teeth, abnormal development of permanent teeth, periodontitis, and severe dental caries with periapical abscess formation. Primary teeth usually fail to exfoliate, which may impair secondary dentition emergence [6]. The retention of primary teeth has been thought to be due to reduced resorption of the tooth roots that result in the failure of eruption of permanent teeth [7]. The extraction of primary teeth usually results in normal eruption of the permanent dentition. Other oral cavity abnormalities have also been described, including a high arched palate, central ridges, and fissures of the palate and deep grooves on the tongue and buccal mucosa with multiple fissures [8]. We present a classic case of a patient with autosomal dominant hyper IgE syndrome and the identification of a rare genetic mutation of the STAT3 gene.

## 2. Case Report

**Figure 1:**
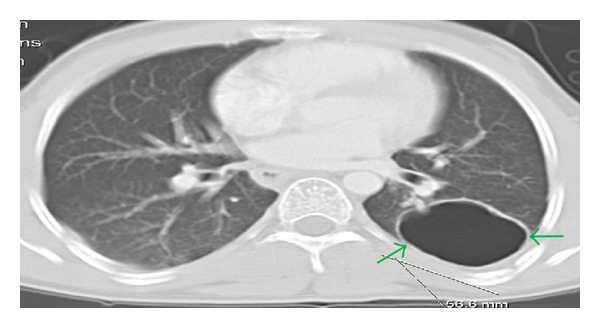
Chest CT: residual 6 cm thin walled cyst in the posterior aspect of the left midlung field (green arrows).

A 23-year-old Hispanic male presented to our care with a sore throat and odynophagia to liquid and solids for 3 days. He denied any fever, chills, nausea, vomiting, diarrhea, cough, or shortness of breath. He claimed that he had frequent respiratory and gastrointestinal infections when he was a child. On a previous hospital admission 2 years ago, he was discovered to have a lung abscesses (*Staphylococcus aureus*) and oral candidiasis. The computed tomography (CT) of the chest on the previous admission revealed a large consolidation involving the right lung and posterior segment of the left lower lobe with an air fluid level. These bilateral lung abscesses were drained and he was treated for aspiration pneumonia with clindamycin 300 mg PO four times a day for one month upon discharge. The CT of the chest (Figure 1) after drainage of the abscess revealed residual 6 cm thin walled cyst in the posterior aspect of the of left midlung field. Other past medical problems included recurrent *Staphylococcus aureus* skin infections. He had no family history of any immunodeficiency disorders. He admitted to smoking 1 pack of cigarettes per week for the last 9 years and social alcohol use. He denied any illicit drugs use.

**Figure 2:**
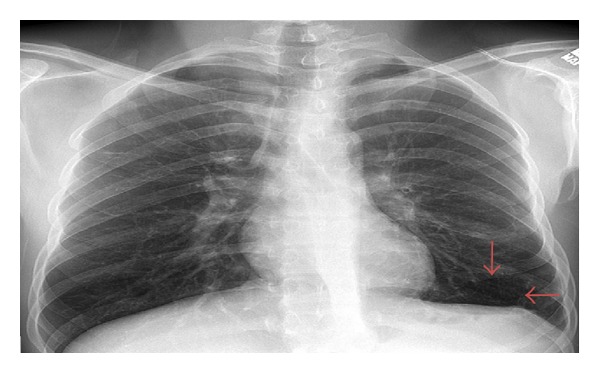
CXR: left lung cyst (red arrows).

Initial vital signs were significant for hypertension (blood pressure: 147/85), tachycardia (heart rate: 116), and tachypnea (respiratory rate: 25 breaths per minute). Physical examination revealed a cachectic individual with multiple eczematous lesions and folliculitis over his entire body. He had white lesions resembling thrush on his tongue, palate, and oropharynx. It was noted that he had retention of his primary teeth. Breath sounds were decreased bilaterally on auscultation. He was tachycardic but without murmurs. He had a decreased muscle mass of all four extremities. The initial lab work-up (Table 1) was only significant for acute renal insufficiency (BUN 25 mg/dL and creatinine 2.2. mg/dL), hypocalcemia (calcium 7.9 mmol/L), hypoalbuminemia (albumin 2.8 g/dL), and hypoproteinemia (7.9 mmol/L). The chest radiograph (Figure 2) on admission revealed a left lung cyst.

Our initial impression was oral/esophageal candidiasis and the possibility of an immunodeficiency disorder. For the treatment of oral/esophageal candidiasis, he was started on fluconazole 100 mg IV daily. The HIV test was negative. Other lab work-ups (Table 2) was ordered to rule out various immunodeficiency disorders. All the immunoglobulins (Ig) were within normal range except that IgE was very elevated (>5000 IU/mL). The normal value of IgE was <114 IU/mL. Genetic mutation analysis revealed a novel mutation affecting the transactivation domain of the STAT3 gene at exon 22. This provided a definite diagnosis of autosomal dominant hyper IgE syndrome which correlates with the clinical presentation. These results confirmed our suspicion of hyper IgE syndrome (Job's syndrome). No curative treatment is available for hyper IgE syndrome. We treated his multiple skin infections with mupirocin 2% ointment and started prophylactic treatment of recurrent skin and pulmonary infections with trimethoprim-sulfamethoxazole 160 mg tab every 12 hours to prevent recurrent staphylococcal infections.

**Figure 3:**
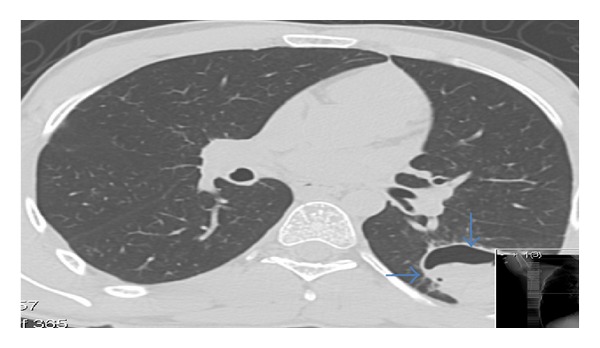
Chest CT: cavitation measuring 5.6 cm in the superior segment of the left lower lobe that contained soft tissue density (blue arrows).

Approximately one year after previous, he presented again with a productive cough with greenish sputum, fever, chills, generalized weakness, eczema, chest pain, and shortness of breath for 4 days. On the day of admission he had 3 bouts of hemoptysis. The CT of the chest (Figure 3) revealed the same cavitation measuring 5.6 cm in the superior segment of the left lower lobe (LLL) that contained soft tissue density that was suggestive of a mycetoma. A bronchoscopy was performed on this admission with findings of hemoptysis and cavitation in the LLL. The analysis of the bronchoalveloar lavage fluid did not reveal any pneumocystis or fundal organisms. He was discharged on amoxicillin-sulbactum 500 mg tab every 12 hours and itraconazole 200 mg tab every 12 hours. Even though the patient had recurrent infections typical of hyper IgE as a child, he never received an immunodeficiency investigation. Partly because hyper IgE is a rare disease that can have a variety of presentations and not all physicians may consider it in their differential diagnosis, another reason as stated by the patient was that he was treated for the infection and then either lost to follow up or went to another hospital once another infection recurred. This case demonstrates the chronic debilitating course of a patient with hyper IgE.

## 3. Discussion

The hallmark of hyper IgE syndrome is an increased concentration of immunoglobulin E (IgE) in the serum. A value of >2000 IU/mL has been considered the cutoff point in establishing a definitive diagnosis of the syndrome [9]. The diagnosis of hyper IgE syndrome is based on characteristic clinical phenotypes associated with increased serum levels of IgE and eosinophilia. In 93% of patients with hyper IgE syndrome will also exhibit peripheral eosinophilia [9]. Eosinophilia is common but does not always correlate with the serum IgE. In approximately 20% of cases, the IgE will normalize during adulthood [10]. Other immunoglobulins are frequently normal, although some may have low serum IgA or slightly low serum IgG. Hyper IgE syndrome is a genetic disorder that is either autosomal dominant or recessive. However, a definite diagnosis can be made with genetic mutation analysis. Autosomal dominant hyper IgE syndrome will have abnormalities in multiple systems, including skeletal/dental, connective tissue, and immune systems. Autosomal dominant hyper IgE syndrome manifests characteristics of coarse facies, skeletal/connective tissue abnormalities (pathological fracture, scoliosis, hyperextensibility, and retention of deciduous teeth), and pneumatocele after pulmonary staphylococcal infection [11].

The majority of autosomal dominant hyper IgE syndrome is sporadic mutations. The autosomal dominant hyper IgE syndrome is associated with a missense or in-frame deletions in the SH2 and DNA-binding domains in the STAT3 gene (Signal Transducer and Activator of Transcription 3) [12]. Woellner et al. found 18 novel mutations in STAT3 gene among a cohort of 100 patients with suspected hyper IgE syndrome [13]. It is deemed necessary to sequence the entire STAT3 gene to exclude possible mutations. Mutations could involve the exon or introns of the DNA-binding domain, SH2 domain, which are more common or the very rarely, and involve the transactivation domain or coiled-coil domain. Patients with the STAT 3 mutation have reduced numbers of interleukin (IL) 17 producing CD4 T cells. Those patients without the STAT3 mutation had a significant reduction of IFN-gamma producing CD4 T cells. Woellner et al. therefore suggest that Th-17 could also be used to distinguish hyper IgE syndrome patients with or without the STAT3 mutation [13]. IL-10 is one of the cytokines that are signaled through STAT3 gene. IL-10 plays an anti-inflammatory role that affects the extent of anti-/proinflammatory cytokine production and the regulation of monocytes [14]. It also inhibits the function of macrophages, suppresses inflammatory cytokines (IL-1, IL-6, IL-8, and TNF-alpha), and inhibits the function of antigen presenting cells (APC) [14].

Autosomal recessive hyper IgE syndrome is associated with a deficiency of tyrosine kinase 2 (TYK2). Tyrosine kinase 2 deficiency is responsible for the impairment of the innate and adaptive immune response due to defective cytokine signal transduction pathways which depend on interferon (IFN)-*α*, IL-6, IL-10, IL-12, and IL-23 [15]. Autosomal recessive hyper IgE syndrome displays abnormalities that are confined to the immune system. Autosomal recessive hyper IgE syndrome presents with severe viral infections, central nervous system involvement, and intracellular bacterial infections, but absence of pneumatocele and skeletal/connective tissue abnormalities [15]. Some cases of autosomal recessive hyper IgE syndrome will have a homozygous mutation of the cytokinesis gene, dedicator of cytokinesis 8 (DOCK8) that leads to a disruptive production of a protein involved in the regulation of actin skeleton [15]. Patients with autosomal recessive hyper IgE syndrome lack the connective tissue and skeletal manifestations, but an increased rate of viral infections (DOCK8 mutations) and intracellular bacteria (TYK2 mutations) [16]. Hematopoietic stem cell transplantation (HSCT) is not considered for hyper IgE syndrome but should be considered in *DOCK8* deficiency [17].

The management of hyper IgE syndrome patients is difficult, mainly because the pathophysiology of the immunodeficiency is not completely understood. No curative treatment is available; however, prophylactic antibiotics, specific treatment depending on the involved organ system, and systemic antibiotics for infections have been suggested. Some authorities also recommend antifungal prophylaxis with itraconazole. A major goal of treatment is aggressive control of skin and sinopulmonary infections. Prophylactic antibiotic therapy with trimethoprim-sulfamethoxazole is recommended in patients with recurrent sinopulmonary and cutaneous infections to prevent Staphylococcal infections. The control of respiratory infections can help decrease the risk of parenchymal lung damage. The main therapeutic approach to hyper IgE syndrome is the prevention and management of infections. Empiric therapy of active respiratory infections with the introduction of antibiotics early on to cover such microorganisms as *Staphylococcus aureus*, Streptococcus pneumonia, and *Haemophilus influenzae* is recommended. Skin or lung abscesses may require surgical intervention.

There are a few studies in the current literature suggesting that intravenous immunoglobulin (IVIg) or hematopoietic cell transplantation (HCT) could be viable options in the treatment of hyper IgE. Wakim et al. reported no dramatic laboratory or clinical improvement with the use of IVIG in the treatment in patients with hyper IgE syndrome. IVIG does not provide a clear clinical benefit to help decrease IgE level [18]. Gennery et al. suggested that hematological and immunological reconstitution with donor bone marrow stem cells does not alter the disease process in hyper IgE syndrome [19]. However, Mcdonald et al. reported on a child with autosomal recessive hyper IgE syndrome (ARHIES) with DOCK8 deficiency that underwent allogeneic hematopoietic cell transplantation (HCT) after myeloablative conditioning and demonstrated full donor chimerism early after transplant [20]. They suggested that HCT could be a viable therapy for these patients. Hematopoietic stem cell transplantation (HSCT) is not considered for hyper IgE syndrome but should be considered in *DOCK8* deficiency. However, very few reports exist regarding the use of the IVIg or HCT for hyper IgE syndrome. Clearly more experience and further research are required to evaluate the clinical outcomes and future of these therapies.

Patients with the hyper IgE syndrome require interdisciplinary care by many specialists. Hyper IgE syndrome is a challenge for clinicians in establishing a diagnosis in suspected cases. Hyper IgE should be considered in the differential diagnosis when presented with a patient that exhibits clinical manifestations as mentioned above. Genetic mutation analysis provides a definitive diagnosis of hyper IgE syndrome. The case presented is very typical of STAT3 deficient autosomal dominant hyper IgE syndrome (ADHIES). The unique finding in this case is the very rare finding of the transactivation domain STAT3 mutation rather than the usual mutation of the SH2 or DNA-binding domain. There is no definitive therapy for hyper IgE syndrome. However, this case demonstrates a chronic debilitating course that requires further investigative research to develop a more effective therapy.

## Figures and Tables

**Table 1:** Initial laboratory work-up.

White blood cell count	10.0 × 10^3^ UL (4.5–11.0 × 10^3^/UL)
Hemoglobin	12.4 g/dL (12.0–15.0 g/dL)
Hematocrit	37.0% (36.0–47.0%)
Platelet count	198 × 10^3^/UL (150–450 × 10^3^/UL)
Sodium	145 mmol/L (135–145 mmol/L)
Potassium	4.3 mmol/L (3.5–5.1 mmol/L)
Chloride	119 mmol/L (98–107 mmol/L)
CO_2_	17 mmol/L (21–32 mmol/L)
Serum glucose	85 mg/dL (70–100 mg/dL)
BUN	25 mg/dL (7–22 mg/dL)
Creatinine	2.2 mg/dL (0.60–1.30 mg/dL)
Calcium	7.9 mmol/L (8.5–10.1 mmol/L)
Albumin	2.8 g/dL (3.4–5.0 g/dL)
Protein	5.9 g/dL (6.4–8.2 g/dL)
AST	29 IU/L (15–37 IU/L)
ALT	19 IU/L (12–78 IU/L)
Alkaline phosphatase	83 IU/L (50–136 IU/L)

**Table 2:** Other laboratory work-ups.

Complement 3 (C3)	113 mg/dL (74–148 mg/dL)
Complement 4 (C4)	35 mg/dL (14–39 mg/dL)
CH 50	52 U/mL (30–75 U/mL)
IgM	84 mg/dL (54–296 mg/dL)
IgD	9 mg/dL (<10 mg/dL)
IgE	>5000 IU/mL (<114 IU/mL)
IgA	158 mg/dL (50–400 mg/dL)
IgG	1480 mg/dL (600–1500 mg/dL)

## References

[1] Esposito L, Poletti L, Maspero C (2012). Hyper-IgE syndrome: dental implications. *Oral Surgery, Oral Medicine, Oral Pathology and Oral Radiology*.

[2] Rael EL, Marshall RT, McClain JJ (2012). The Hyper-IgE syndromes: lessons in nature, from bench to bedside. *World Allergy Organization Journal*.

[3] Minegishi Y (2009). Hyper-IgE syndrome. *Current Opinion in Immunology*.

[4] Montella S, Maglione M, Giardino G (2012). Hyper IgE syndrome presenting as chronic suppurative lung disease. *Italian Journal of Pediatrics*.

[5] Zhang Q, Su HC (2011). Hyperimmunoglobulin e syndromes in pediatrics. *Current Opinion in Pediatrics*.

[6] Freeman AF, Holland SM (2010). Clinical manifestations of hyper IgE syndromes. *Disease Markers*.

[7] Freeman AF, Domingo DL, Holland SM (2009). Hyper IgE (Job’s) syndrome: a primary immune deficiency with oral manifestations. *Oral Diseases*.

[8] Yong PFK, Freeman AF, Engelhardt KR, Holland S, Puck JM, Grimbacher B (2012). An update on the hyper IgE syndromes. *Arthritis Research & Therapy*.

[9] Koslovsky DA, Kostakis VA, Glied AN, Kelsch RD, Wiltz MJ (2013). An unusual lesion of the tongue in a 4-year-old with Job syndrome. *Journal of Oral and Maxillofacial Surgery*.

[10] Heimall J, Freeman A, Holland SM (2010). Pathogenesis of hyper IgE syndrome. *Clinical Reviews in Allergy and Immunology*.

[11] Liu J-Y, Li Q, Chen T-T, Guo X, Ge J, Yuan L-X (2011). Destructive pulmonary staphylococcal infection in a boy with hyper-IgE syndrome: a novel mutation in the signal transducer and activator of transcription 3 (STAT3) gene (p.Y657S). *European Journal of Pediatrics*.

[12] Roxo P, Menezes UP, Tucci S, Andrade MF, Barros Silva GE, Lima Melo JM (2013). Renal abscess in hyper-IgE syndrome. *Urology*.

[13] Woellner C, Gertz EM, Schäffer  AA (2010). Mutations in STAT3 and diagnostic guidelines for hyper-IgE syndrome. *The Journal of Allergy and Clinical Immunology*.

[14] Giacomelli M, Tamassia N, Moratto D (2011). SH2-domain mutations in STAT3 in hyper-IgE syndrome patients result in impairment of IL-10 function. *European Journal of Immunology*.

[15] Szczawinska-Poplonyk A, Kycler Z, Pietrucha B, Heropolitanska-Pliszka E, Breborowicz A, Gerreth K (2011). The hyperimmunoglobulin E syndrome—clinical manifestation diversity in primary immune deficiency. *Orphanet Journal of Rare Diseases*.

[16] Rezaei N, Aghamohammadi A (2010). Hyper-IgE syndrome. *Journal of Postgraduate Medicine*.

[17] Roxo P, Torres LAGM, Menezes UP, Melo JML (2013). Lung function in hyper IgE syndrome. *Pediatric Pulmonology*.

[18] Wakim M, Alazard M, Yajima A, Speights D, Saxon A, Stiehm ER (1998). High dose intravenous immunoglobulin in atopic dermatitis and hyper-IgE syndrome. *Annals of Allergy, Asthma and Immunology*.

[19] Gennery AR, Flood TJ, Abinun M, Cant AJ (2000). Bone marrow transplantation does not correct the hyper IgE syndrome. *Bone Marrow Transplantation*.

[20] Mcdonald DR, Massaad MJ, Johnston A (2010). Successful engraftment of donor marrow after allogeneic hematopoietic cell transplantation in autosomal-recessive hyper-IgE syndrome caused by dedicator of cytokinesis 8 deficiency. *Journal of Allergy and Clinical Immunology*.

